# Treatment choices for managing glucose control in impaired renal function: Interim Results

**DOI:** 10.23889/ijpds.v1i1.393

**Published:** 2017-04-19

**Authors:** Gareth Davies, Jeff Stephens, Sam Rice, James Chess

**Affiliations:** 1 Swansea University; 2 NHS Wales

## Objectives

Patients with chronic kidney disease (CKD ≥3) and diabetes mellitus comprise approximately 25% patients with diabetes. These patients are at a higher risk of cardiovascular morbidity and mortality and furthermore therapies targeting glucose control are limited. The management of glycaemic control in type 2 diabetes and chronic renal disease is difficult with limited therapeutic choices. This issue has been a matter of longstanding debate. Following a number of joint Diabetes-Renal meetings between the Diabetes and Renal teams based in Hywel Dda and ABM University Health Boards, a proposal was put forward to the SAIL team to examine the relationship between diabetes therapies in relation to eGFR, as this may influence further practice and guidance for patients with type 2 diabetes and renal impairment.

## Approach

Linkage and re-use of routinely collected anonymised clinical data held in the SAIL databank was employed, to identify a cohort of adult patients in Abertawe Bro Morgannwg Health Board (ABMU) having type 2 diabetes (excluding type 1 diabetes). Diagnosis of diabetes was achieved by use of National Health Service ‘Read’ codes. Creatinine, eGFR, age, gender, weight, height, cholesterol, LDL, HDL, TG, systolic blood pressure, diastolic blood pressure, diabetes medication prescriptions, the use of statins, ACEis, aspirin, CHD status, CVD status, duration of diabetes were identified in primary care GP and pathology datasets.

## Results

42170 (6.0%) of adults in ABMU were identified as having type 2 (excluding type 1) diabetes , 13369 of which had good GP registration coverage. The gender split was male 56%, female 44%. Duration of diabetes (years) was (mean/median/SD/IQR) 9.96/8.97/6.78/8.10; weight (Kg) was 86.94/85.00/21.23/28.19; age (years) 65.49/66.74/13.75/19.03; BMI 31.57/30.70/6.62/7.99. Incidence of CKD as defined by GP coded data was 24%, renal replacement therapy 0.4%, Ischaemic Heart Disease 22%. Prevalence of prescriptions during 2014 was: Anti-diabetic medication 72%, statins 75%, aspirin 34%, ACEi/ARB 61%. The import of pathology laboratory data into SAIL is currently pending, and is anticipated before April 2016. This will allow the accurate stratification of CKD status and detailed description of use of anti-diabetic agents.

## Conclusion

The project methods and coding structure are well place to provide anticipated results as soon as pathology data arrives. The percentage of ABMU patients having type 2 diabetes is in line with other literature for adults in the UK.

